# Murmur identification and outcome prediction in phonocardiograms using deep features based on Stockwell transform

**DOI:** 10.1038/s41598-024-58274-6

**Published:** 2024-03-31

**Authors:** Omid Dehghan Manshadi, Sara mihandoost

**Affiliations:** Department of Electrical Engineering, Urmia University of Technology, Urmia, Iran

**Keywords:** Phonocardiogram (PCG), Stockwell transform, CNN, CinC/physionet 2022 dataset, Biomedical engineering, Data processing, Machine learning

## Abstract

Traditionally, heart murmurs are diagnosed through cardiac auscultation, which requires specialized training and experience. The purpose of this study is to predict patients' clinical outcomes (normal or abnormal) and identify the presence or absence of heart murmurs using phonocardiograms (PCGs) obtained at different auscultation points. A semi-supervised model tailored to PCG classification is introduced in this study, with the goal of improving performance using time–frequency deep features. The study begins by investigating the behavior of PCGs in the time–frequency domain, utilizing the Stockwell transform to convert the PCG signal into two-dimensional time–frequency maps (TFMs). A deep network named AlexNet is then used to derive deep feature sets from these TFMs. In feature reduction, redundancy is eliminated and the number of deep features is reduced to streamline the feature set. The effectiveness of the extracted features is evaluated using three different classifiers using the CinC/Physionet challenge 2022 dataset. For Task I, which focuses on heart murmur detection, the proposed approach achieved an average accuracy of 93%, sensitivity of 91%, and F1-score of 91%. According to Task II of the CinC/Physionet challenge 2022, the approach showed a clinical outcome cost of 5290, exceeding the benchmark set by leading methods in the challenge.

## Introduction

The prevalence of cardiovascular diseases remains a leading global cause of mortality, constituting about one-third of all recorded deaths worldwide ^1^. This issue is particularly critical in low-income countries, where healthcare systems face substantial challenges. Identifying and treating acquired and congenital heart conditions present formidable obstacles due to the scarcity of specialized cardiologists in remote and underprivileged areas with limited access to healthcare ^2,3^. Consequently, a vast majority of patients in these settings lack access to consultations with qualified cardiologists.

A digital cardiac examination provides an affordable and straightforward method for capturing heart sounds at various crucial points without extensive training ^4^. In spite of this, interpreting these sounds still requires significant and prolonged training ^5,6^. Automated detection and interpretation of PCGs is gaining traction as a way to overcome the limitations of manual examination of heart sounds, which requires extensive training. The automated examination of the heart enables the early detection of congenital and acquired diseases, especially in children, by examining the heart's mechanical function without invasive procedures.

In the last two decades, significant research efforts have been dedicated to automating heart disorders diagnosis by leveraging PCG signals and artificial intelligence (AI) techniques. Noteworthy studies in PCG signal classification showcase diverse methodologies. El Badlaoui et al., applied principal component analysis (PCA) alongside a support vector machine (SVM) classifier ^7^. They experimented with various hyperparameters and kernels, employing this approach on two distinct private PCG datasets. Sawant et al. introduced a technique utilizing wavelet transform (WT) and gradient boosting (GB), achieving a notable 90.25% accuracy on both the PASCAL and the Computing in Cardiology Challenge (CinC) /Physionet challenge 2016 datasets ^8^. Abduh et al. utilized a mel-frequency coefficients (MFCC) along with fractional Fourier transform, employing k-nearest neighborhoods (KNN) and SVM analysis on the CinC /Physionet challenge 2016 dataset ^9^. Wu et al. classified PCG recordings using MFCC and the hidden Markov model (HMM) ^10^. Maglogiannis et al. employed WT and morphological analysis, integrating an SVM classifier on a representative global dataset ^11^. Li et al. classified heart sounds using fractals WT and SVM, focusing on the CinC /Physionet challenge 2016 dataset ^12^. Rujoie et al. used MFCC and Hilbert transform (HT) for feature extraction, coupling it with KNN for PCG classification on a private database ^13^. Chen et al. applied S transforms, along with features based on discrete time–frequency energy, to classify heart sounds in a private dataset^14^. Additionally, in references ^14–17^, authors extracted time–frequency features through synchrosqueezing, polynomial chirplet transform, and spline chirplet-based methods from PCG signals, employing diverse classifiers for PCG signal classification.

Moreover, several studies have utilized different techniques based on deep learning (DL) for PCG classification. Singh et al. utilized 2D scalograms with continuous WT (CWT) and a convolutional neural network (CNN) on the CinC /Physionet challenge 2016 dataset ^18^. Baghel et al. employed a six-layer CNN for heart valve disorder (HVD) detection using PCG signals ^19^. Furthermore, Alkhodari and Fraiwan implemented a convolutional recurrent neural network (RNN)-based model to identify various types of HVDs ^20^. Soares et al. used a neuro-fuzzy based modeling approach, combining CNN with different classifiers, achieving 93% accuracy on the CinC /Physionet challenge 2016 dataset ^21^. Li et al. introduced a fusion framework model incorporating multi-domain features and deep learning features extracted from PCG ^22^. Bozkurt et al. conducted research on time–frequency features combined with a deep model for heart sound classification, achieving an accuracy of 86.02% on the CinC /Physionet 2016 dataset ^23^.

A substantial portion of the existing research in this domain heavily relies on the CinC /Physionet challenge 2016 dataset ^2^, which were made available as part of a specific challenge. However, it's essential to recognize that these datasets primarily rely on the binary classification of PCG signals. Additionally, a significant limitation is observed in the analysis of different heart sound samples from the same patient independently, without considering their common source. This oversight disregards the potential benefits of leveraging multiple sounds from a single patient to enhance diagnostic accuracy. This is done by taking into account the varying intensity of murmurs across different auscultation locations^24^. The introduction of the CinC /Physionet challenge 2022 dataset ^25^ marks a significant step in addressing certain limitations prevalent in current heart sound datasets. In addition to binary labels, this dataset introduces a novel unknown label. Notably, it presents multiple heart sound recordings from diverse auscultatory locations for each patient. This multifaceted feature opens up new avenues for leveraging this data to achieve more precise diagnostics.

Several research teams have recently developed distinct algorithms aimed at distinguishing between the murmur presence, absence, and uncertain cases within multi-location PCGs, as part of the CinC /Physionet challenge 2022. These teams underwent evaluation using a weighted accuracy metric for Task I and a cost-based scoring metric for Task II ^25^. The top three algorithms in Task I are documented as references ^26–28^. Lu et al. proposed a combination of the mel-spectrogram and various wide features as inputs for a CNN, resulting in an 80% accuracy on the CinC /Physionet challenge 2022 dataset ^26^. McDonald et al. utilized hidden semi-Markov models and RNN to detect murmurs and perform reliable PCG segmentation with an accuracy of 85% ^27^. Finally, Xu et al. proposed a CNN-based approach, where spectrograms at different scales were computed and combined into a single CNN, achieving an accuracy of 90% ^28^.

In our study, we introduce a new hybrid model to represent PCG signals by combining the Stockwell transform with a DL technique. The Stockwell transform generates a time–frequency map (TFM) of the PCG, which is used for feature extraction using AlexNet. These extracted features are fed into different classifiers for murmur detection. Our experimental results demonstrate that our proposed method outperforms the leading techniques from the CinC /Physionet challenge 2022, highlighting its outstanding performance.

The paper is organized as follows in the subsequent sections: The section entitled "Materials and Methods" elucidates the dataset information and the proposed methodology for the study. Following that, the section titled "Results" presents the outcomes of the performance assessment. Finally, the paper delves into a discussion of these results. It concludes with the section "Discussion and Conclusion," which summarizes the main findings and provides a final overview of the study.

## Material and method

**Figure 1:**
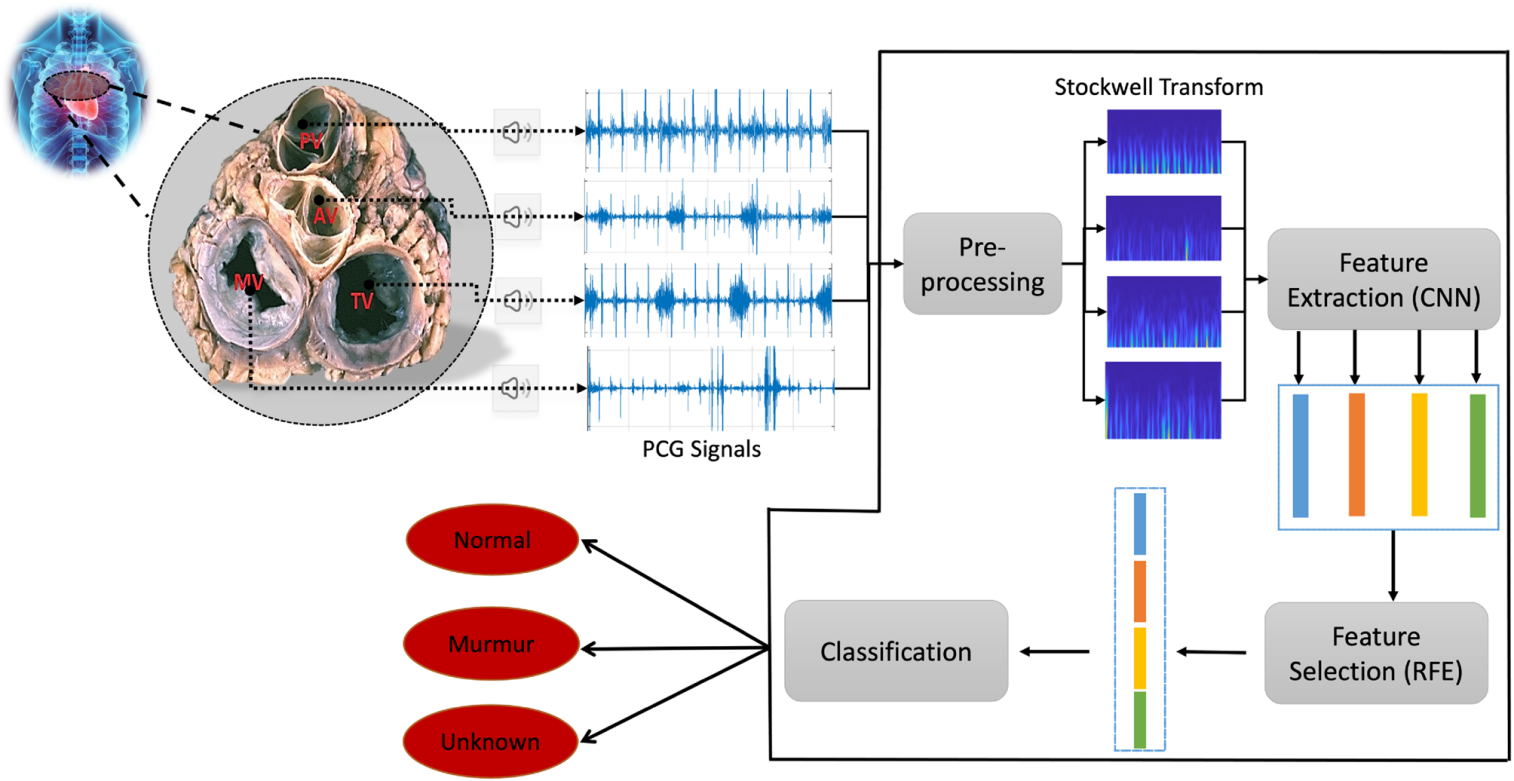
Illustrates the pipeline representing the proposed method for PCG classification, where RFE stands for Recursive Feature Elimination and CNN represents Convolutional Neural Network.

This section presents the dataset we used in our study and describes our approach to classifying PCG signals. A visual representation of our method is shown in Fig. 1. It consists of four steps: (I) time–frequency analysis using the Stockwell transform, (II) extraction of deep features using the AlexNet model, (III) reducing dimensionality and (IV) classification. Each of these steps will now be explained in detail.

### The PCG database

The dataset used in this study, the CinC /Physionet challenge 2022 dataset ^29,30^, constitutes a considerable compilation of PCG signals collected from pediatric subjects in Brazil between 2014 and 2015. This dataset comprises 3,163 audio files from 942 patients, recorded at a sampling rate of 4,000 Hz. The files have durations between 5 and 65 s. Included within the dataset are multiple PCG recordings obtained from diverse auscultation locations, including the aortic valve (AV), pulmonary valve (PV), tricuspid valve (TV), and mitral valve (MV). While most patients have recordings at all four locations, some patients have fewer recordings. A few possess multiple recordings at each location. Notably, the recordings were obtained sequentially resulting in variations in the number, location, and duration of recordings among patients ^24^. The dataset underwent meticulous annotation by an expert annotator, categorizing each record as “murmur present” (2391 recordings), “absent” (616 recordings), or “unknown” (156 recordings, indicative of low-quality records). Additionally, the data was classified into two classes: normal (2575 recordings) and abnormal (665 recordings). For more detailed information about the dataset, refer to reference ^29^. It is imperative to note that the Physionet Challenge 2022 comprises two separate tasks. In Task I, PCG signals were classified into three different classes based on the initial categorization. In Task II, the objective was to identify normal and abnormal patients using the second categorization scheme.

### Time–frequency analysis using Stockwell transform

Due to the nonlinearity and non-stationarity behavior of PCG signals, different time–frequency transformations, such as the wavelet and chirplet transform, have traditionally been used for PCG analysis ^8^. However, the wavelet transform has limitations, including the necessity to select an appropriate mother wavelet and account for the loss of absolute phase information in the data. Hence, in this approach, we employed the Stockwell transform to depict PCG records in the time–frequency domain. This transform ^31^, denoted as ${S}_{z}\left(\tau ,f\right)$, for a continuous time signal $z(t)$, is formulated as follows:

1 $${S}_{z}\left(\tau ,f\right)={e}^{j2\pi \tau }{W}_{z}(\tau ,d)$$

where $d$ represents the inverse of frequency ($d=1/f$). Additionally, ${W}_{z}\left(\tau ,d\right)$ denotes the continuous wavelet transformation of the signal $z(t)$, using the Gaussian mother wavelet:

2 $${W}_{z}\left(\tau ,d\right)={\int }_{-\infty }^{+\infty }z\left(t\right)w(t-\tau ,d)dt$$

3 $$w\left(t,f\right)=\frac{\left|f\right|}{\sqrt{2\pi }}{e}^{\frac{-{t}^{2}{f}^{2}}{2}}{e}^{-j2\pi ft}$$

Therefore, Eq. (1) is modified as:

4 $${S}_{z}\left(\tau ,f\right)={\int }_{-\infty }^{+\infty }z\left(t\right)\frac{\left|f\right|}{\sqrt{2\pi }}{e}^{\frac{-{(\tau -t)}^{2}{f}^{2}}{2}}{e}^{-j2\pi ft}dt$$

As depicted in Eq. (4), the window width in the Stockwell transform is frequency-dependent, expanding as the frequency decreases and contracting as the frequency increases ^31^. For discrete-time signals, the discrete Stockwell transform is computed using the discrete Fourier transform (DFT). The N-point DFT of the discrete-time signal $z[nT]$ can be formulated as follows:

5 $$Z\left[\frac{k}{NT}\right]=\frac{1}{N}\sum_{n=0}^{N-1}z\left[nT\right]{e}^{\frac{-j2\pi kn}{N}},\quad k=\mathrm{0,1}\dots ,N-1$$

The discrete Stockwell transform is essentially a projection of a vector, which is determined by the time series z[nT], onto a spanning set. Each basis vector is divided by N Gaussian shifted into N local vectors in such a way that the sum of these N local vectors recreates the original basis vector. Consequently, the discrete Stockwell transform for the discrete signal at time z[nT] is defined:

6 $${S}_{z}\left[mT,\frac{n}{NT}\right]=\sum_{k=0}^{N-1}z\left[\frac{k+n}{NT}\right]{e}^{\frac{-2{\pi }^{2}{k}^{2}}{{n}^{2}}}{e}^{\frac{-j2\pi kn}{N}}, \quad n,m=\mathrm{0,1},2,\dots ,N-1$$

The function ${e}^{\frac{-2{\pi }^{2}{k}^{2}}{{n}^{2}}}$ in the Eq. (6) represents the Gaussian function. The Stockwell transform produces a complex-valued function, where the amplitude of the Stockwell response is derived using the following relationship, which considered the basis for feature extraction in our study.

7 $$\left|{S}_{z}\right|=\sqrt{{(Rel\{{S}_{z}\})}^{2}+{(Im\{{S}_{z}\})}^{2}}$$

**Figure 2:**
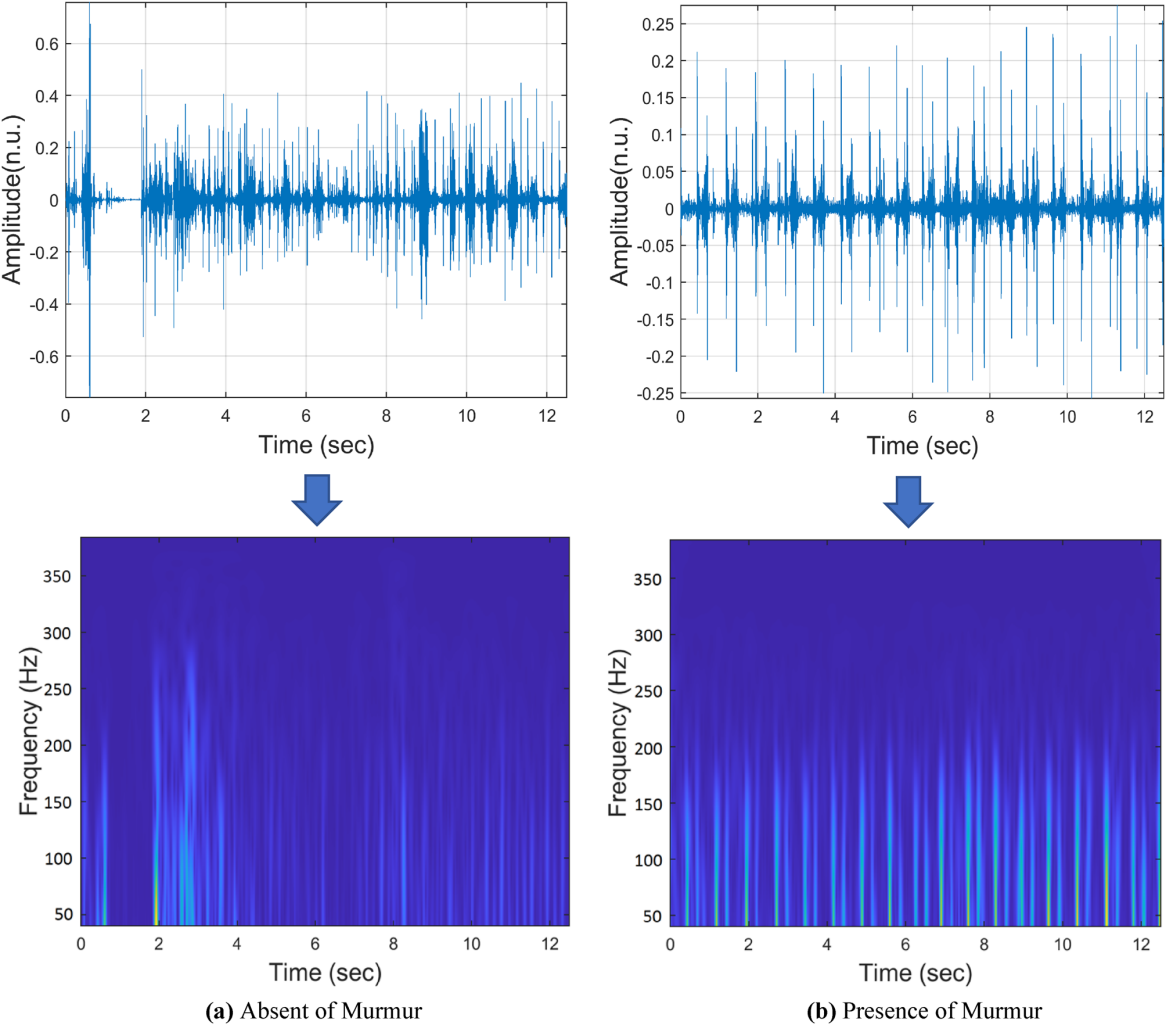
PCG examples in time domain and their corresponding absolute of Stockwell TFM. In the time domain, n.u. indicates normalized units.

Figure 2 illustrates a time–frequency map (TFM) of two PCG signals, one with and one without a murmur. In the presence and absence of a murmur, TFMs show distinct differences between PCG signals. PCG signals, including murmurs, exhibit higher-frequency components on TFMs compared to normal PCG signals. When murmurs are present, this variance may indicate irregularities in the PCG signal, which makes TFMs useful for murmur detection.

### Deep features with Alexnet

**Figure 3:**
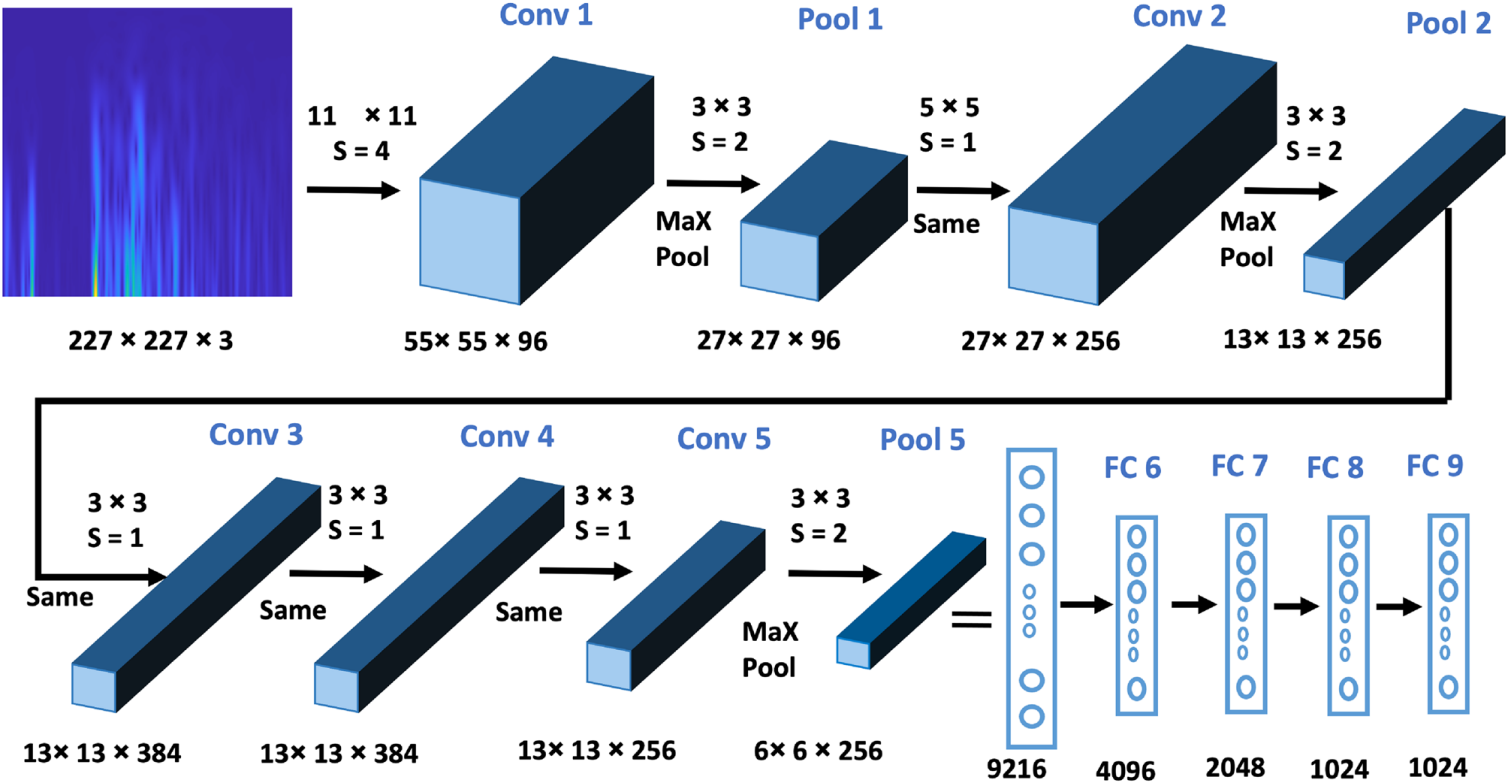
The structure of the used Alexnet.

In our study, we leverage the capabilities of a deep convolutional neural network, AlexNet ^32^, to extract deep features from the prepared TFMs derived from PCG signals. AlexNet could capture intricate patterns within TFMs due to its effective architecture. AlexNet initiates its architecture with an initial convolutional layer (Conv) including 96 filters of size 11 × 11 and a stride of 4 × 4, accompanied by a Rectified Linear Unit (ReLU) activation function. Following each convolutional layer, a 3 × 3 max-pooling layer (Pool) with a stride of 2 × 2 is employed to progressively downscale the TFMs and extract increasingly complex features. This process is visually illustrated in Fig. 3. Subsequent convolutional layers further process the TFMs of varying sizes and strides, aimed at extracting nuanced and intricate features from the data. Once the convolutional and pooling stages are completed, the resulting features are passed through a flatten layer, transforming them into a one-dimensional vector. The model consists of several fully connected (FC) layers, which are crucial to the processing of the extracted data.

### Feature reduction

Following the extraction of deep features, the input TFM transforms into a high-dimensional vector. Some of these features may lack informativeness and exhibit high correlations with each other. To address this, RFE and PCA are employed to reduce the feature vector's dimensionality and choose the most meaningful features.

- *Recursive feature elimination (RFE):* is a feature selection method commonly used in classification problems. It aims to improve the generalization performance of the classification model by iteratively removing the least important features ^33^ . In the context of RFE, the weight vector ($W$) of a linear support vector machine (SVM) is calculated, and the least unimportant feature is determined based on the smallest weight value in $W$. By eliminating these features, RFE seeks to reduce overfitting and enhance classification accuracy ^33^.
- *Principal Component Analysis (PCA):* is a well-known statistical method used to simplify and extract key information from complex data with multiple variables. It seeks to identify a group of perpendicular vectors called principal components that capture the most significant variations in the data. By projecting the data onto these principal components, PCA converts the original high-dimensional data into a lower-dimensional form while retaining essential patterns and structures. The principal components are determined by analyzing the eigenvectors and eigenvalues of the covariance matrix. The eigenvectors signify the directions of maximum variance, while the eigenvalues quantify the variance explained by each principal component ^34^.

### Classification

In this study, three widely recognized machine learning classifiers were employed for PCG classification, and their outcomes were compared. These classifiers are as follows:

*Support Vector Machine (SVM):* It is known for its robust classification capabilities. It's preferred for its reduced computational complexity and suitability for managing small datasets. SVM works by identifying an optimal hyperplane that maximizes the margin between different classes ^35^. In this research, a linear SVM was utilized.

- *Gradient Boosting (GB):* An influential ensemble learning method, which builds a predictive model through the sequential fusion of numerous weak learners. By continually refining its accuracy through a focus on misclassified data points, this technique proves adept at managing intricate datasets and delivering strong predictive capabilities ^36^.
- *Random Forest (RF):* It is a well-known ensemble machine learning classifier ^37^. RF classifiers gather decisions from multiple decision tree (DT) classifiers. RF creates an ensemble of decision trees, each trained on a different subset of features. It aggregates their decisions to improve overall classification accuracy and generalization to new data.

### Tackling data imbalance

To address class imbalance during training, we conducted experiments using resampling techniques, specifically SMOTE ^38^. From our preliminary findings, it seems that employing SMOTE, along with a combination of up-sampling the minority class and down-sampling the majority class, resulted in the highest performance.

## Results

### Metrics

To evaluate the performance of our models, we utilized various evaluation metrics recommended by the PhysioNet Challenge 2022^39^. For the murmur detection task (Task I), the proposed metric is weighted accuracy (WAcc), defined as follows:

8 $$WAcc=\frac{ 5{N}_{PP}+3{N}_{UU}+{N}_{AA} }{5\sum_{i=P,A,U}{N}_{iP}+3\sum_{i=P,A,U}{N}_{iU}+\sum_{i=P,A,U}{N}_{iA}}$$

where P, A, and U denote the presence of a murmur, absence of a murmur, and the unknown class, respectively. For instance, ${N}_{PA}$ indicates the number of patients predicted by the model to have a murmur (presence of murmur) while identified as not having a murmur (absent of murmur) by the expert.

For clinical outcome identification (Task 2), the PhysioNet Challenge 2022 recommended a cost-based scoring metric. This metric takes into account the costs associated with human diagnostic screening, as well as the costs of timely, delayed, and missed treatments ^25^. It is crucial to emphasize that smaller values of this metric are desirable.

9 $${c}_{outcome}=\frac{1}{N}(35N+397M-1718\frac{{M}^{2}}{N}+11296\frac{{M}^{4}}{{N}^{3}}+10000TP+50000FN)$$

Here, N represents the total number of patients, while M represents the number of patients the model recognized as abnormal. This is regardless of whether the prediction was correct or false. TP denotes the number of patients that both the model and the expert correctly identified as abnormal. FN indicates the number of patients the model falsely predicted as normal. Additionally, we evaluated the proposed method's performance using total accuracy (Acc), sensitivity (SE), specificity (SP), and F-score. Furthermore, we employed receiver operating characteristic (ROC) analysis and computed the area under the ROC curve (AUC)^41^.

### Data preparation and feature extraction

In this study, data preparation involved several steps. Initially, we considered a signal duration of 12.5 s, following the reference ^42^ recommendation. To achieve this, we truncated longer records and repeated shorter records, ensuring a consistent duration of 12.5 s for all records. To optimize processing efficiency, we applied a down-sampling technique, reducing the signal's sample rate to 1000. Our focus was specifically on the Stockwell transform output within the frequency band of 20–350 Hz. This was a choice made after experimenting with various frequency ranges. This meticulous selection aimed to optimize the model’s sensitivity while mitigating unnecessary computational costs associated with less informative frequency bands, especially given the infrequent occurrence of murmurs at higher frequencies ^42^. The Stockwell transform was applied to each PCG recording (PV, MV, TV, and AV recordings) for each patient, generating respective TFMs. These TFMs were then individually subjected to AlexNet for deep feature extraction. They amalgamated the extracted deep features from each segments into a single feature vector for each patient. This consolidated feature vector underwent feature reduction algorithms for further analysis and processing. It is crucial to emphasize that the proposed method leveraged all PV, MV, TV, and AV records to provide a comprehensive depiction of each individual patient. No records pertaining to a single patient were incorporated into both training and testing procedures concurrently. Specifically, during the training phase, 80% of patient records were designated for training, while the remaining 20% were reserved for testing. This ensures the model encounters previously unseen data during testing. The proposed method was executed using Google Colab (T4 GPU), with the training process taking approximately 1 h and 37 min and 17 s.

### Classification results

In this section, our objective is to demonstrate the efficiency of combining the Stockwell transform and deep networks for representing PCG data. To assess this, we apply the extracted features to various classifiers and analyze their performance in terms of WAcc, Acc, SE, and SP. This is presented in Table 1. The results obtained from these experiments indicate that the extracted features consistently exhibit good performance across all evaluation metrics when used with all classifiers. This suggests that the extracted features are highly effective at representing PCG data time–frequency characteristics, regardless of the classifier type. Furthermore, it is worth mentioning that while both RF and GB classifiers demonstrate strong performance, RF notably outperforms the other classifiers in similar scenarios.

**Table 1:** Comparing the performance of the various classifiers without feature reduction.

	Acc	WAcc	SE(M)	SE(N)	SE(U)	SP(M)	SP(N)	SP(U)
SVM	0.88	0.87	0.97	0.65	1.0	0.76	0.96	0.93
RF	0.95	0.94	0.92	0.94	0.97	0.93	0.91	1.0
GB	0.92	0.91	0.85	0.92	0.97	0.91	0.85	0.99

### Effect of feature reduction on accuracy

Since not all extracted features are informative and many are redundant, we employed and compared the performance of two distinct techniques, namely PCA and RFE.

**Figure 4:**
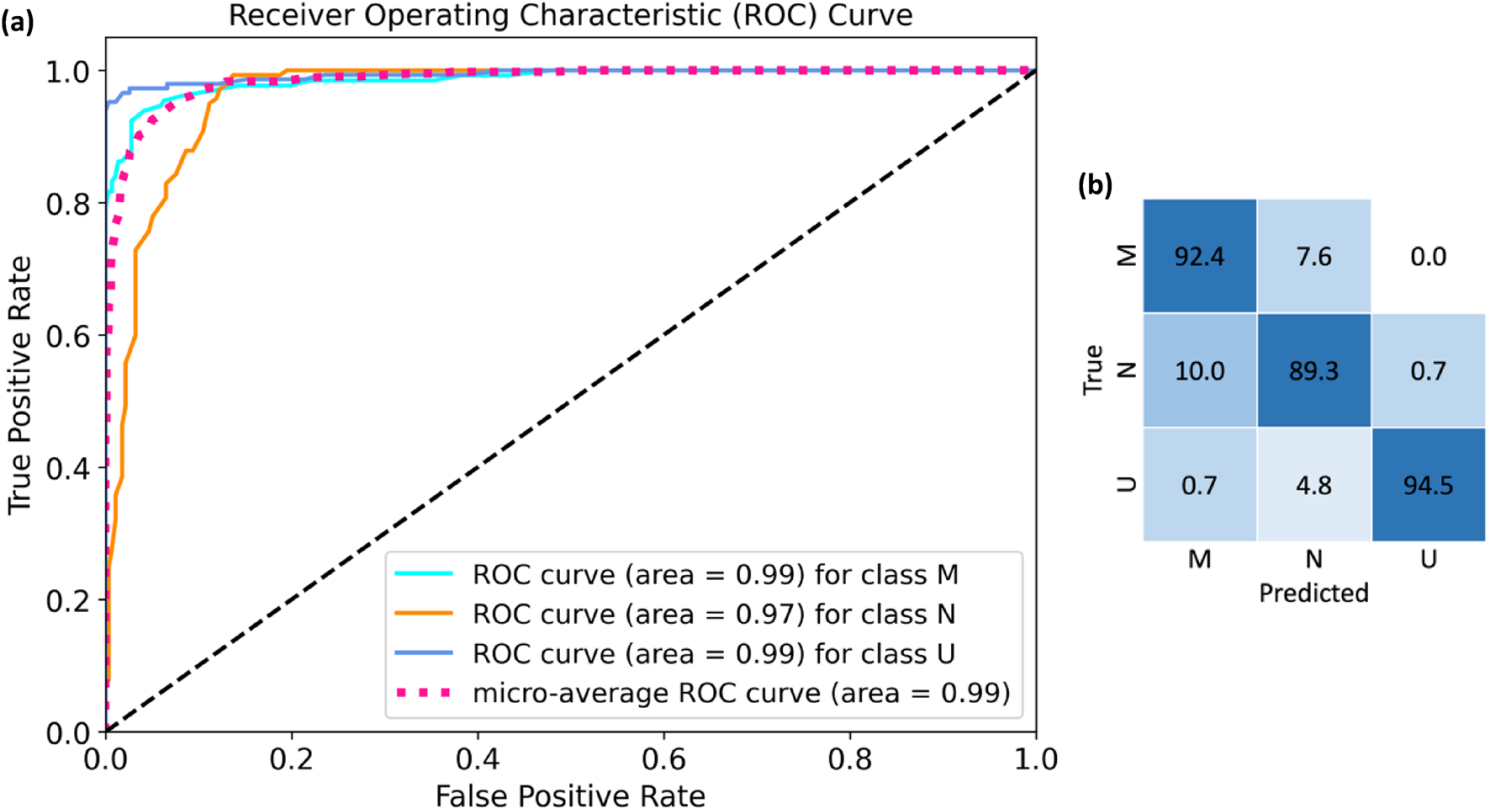
ROC curves and confusion matrix for different classes obtained from feature selection by RFE and RF classification for murmur detection (Task I). (**a**) ROC curves, (**b**) confusion matrix.

**Figure 5:**
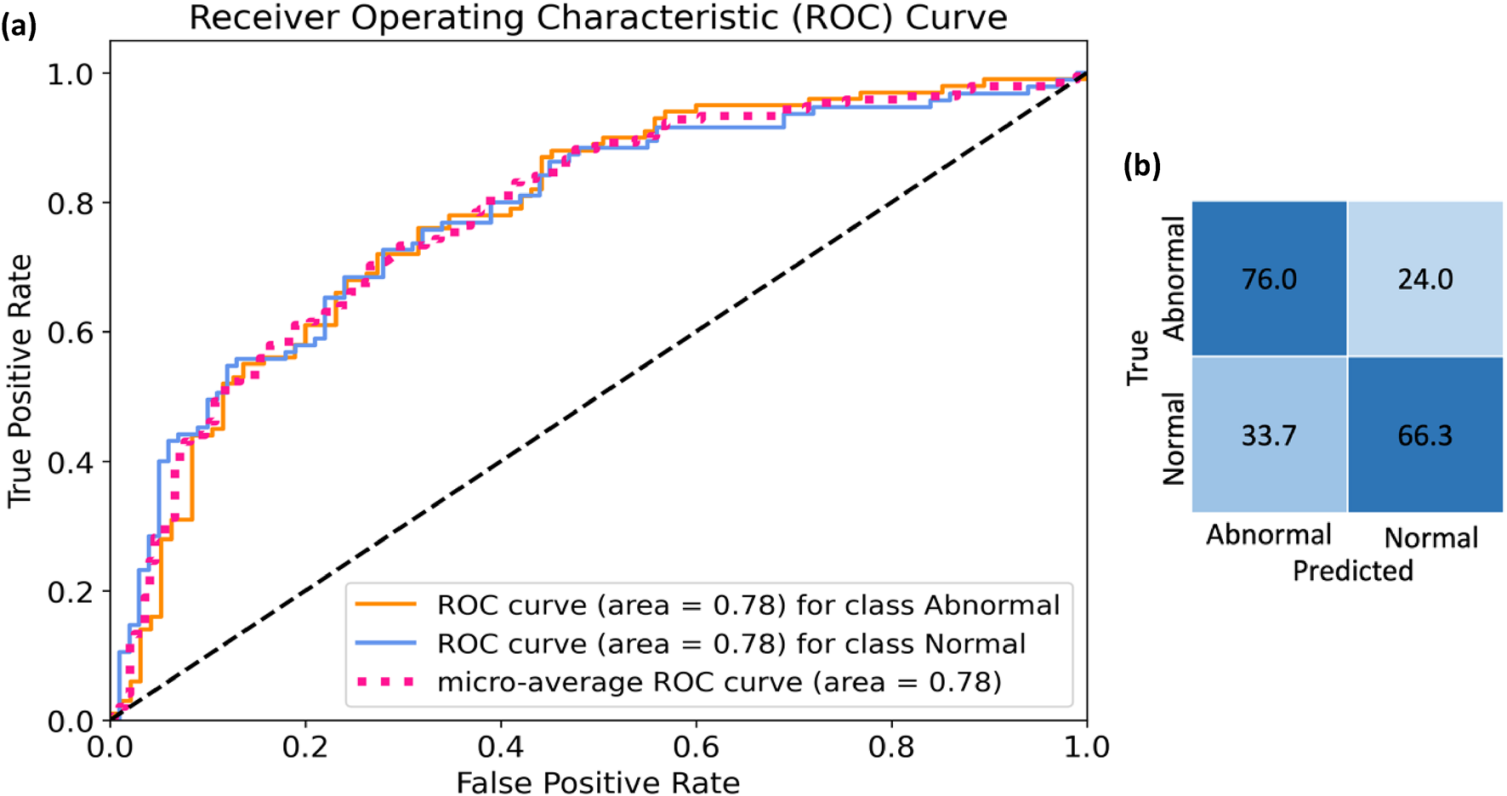
ROC curves and confusion matrix for different classes obtained from feature selection by RFE and SVM classification for clinical outcome prediction (Task II). (**a**) ROC curves, (**b**) confusion matrix.

This was done to reduce the feature vector’s dimensionality. We selected varying numbers of the most significant features, chosen by PCA and RFE. We compared the performance of different classifiers using different feature subsets, as presented in Table 2. Results demonstrate that both feature reduction methods, PCA and RFE, exhibit promising performance across Task I when choosing 120, 240, and 500 features. However, in Task II, RFE outperforms PCA in terms of the weighted accuracy scoring metric (WAcc). Table 2 shows that while RFE-selected 240 and 500 features showed improved performance in Task II, computational complexity and the slight increase in WAcc should be carefully considered. Consequently, the choice of 120 features strikes a good balance. Considering the outcomes outlined in Table 2, we opted for the combination of RFE and RF for Task I and RFE with SVM. We employed 120 features for its superior performance in both tasks. Moreover, for a comprehensive analysis of the selected features and the chosen classifier, additional evaluation metrics have been included. Figure 4 illustrates the results of RF classification based on the top 120 ranked features obtained from RFE. It showcases the ROC curve and confusion matrix for each class separately. Notably, among the AUC values, a 0.99 AUC underscores the remarkable effectiveness of murmur detection. Additionally, Table 3 presents various evaluation metrics such as Acc, SE, SP, and AUC attained by the RFE_RF for Task I. Correspondingly, mirroring Task I, Fig. 5 displays Task II's ROC curve and confusion matrix, while Table 4 outlines diverse evaluation metrics achieved by RFE_SVM for clinical outcome prediction (Task II).

**Table 2:** Performance Comparison of Proposed Method for Different Numbers of Features in Task I and Task II (in terms of WAcc).

		# Features									
		RFE					PCA				
		10	60	120	240	500	10	60	120	240	500
WAcc (Task I)	SVM	0.35	0.62	0.76	0.87	0.91	0.36	0.55	0.70	0.81	0.83
	RF	0.52	0.86	0.93	0.94	0.93	0.73	0.94	0.94	0.94	0.95
	GB	0.48	0.80	0.86	0.86	0.88	0.63	0.82	0.90	0.90	0.92
WAcc (Task II)	SVM	0.46	0.65	0.71	0.73	0.79	0.49	0.42	0.42	0.51	0.49
	RF	0.56	0.55	0.55	0.49	0.52	0.55	0.42	0.42	0.51	0.46
	GB	0.55	0.54	0.60	0.54	0.47	0.49	0.44	0.44	0.47	0.46

**Table 3:** Different evaluation metric obtained by RFE-RF for murmur detection (Task I).

Classifier	Acc (%)	SE (%)	SP (%)	F-score (%)	AUC (%)
RF	92.1	91	91	91	98

**Table 4:** Different evaluation metric obtained by RFE-SVM for clinical outcome prediction (Task II).

Classifier	Acc (%)	SE (%)	SP (%)	F-score (%)	AUC (%)
SVM	71.3	66	76	69	78

### Performance comparison

Table 5 presents the outcomes derived from the proposed method. It compares with the validation scores of the five best entries in the 2022 CinC/PhysioNet challenge in Task I. The results highlight the superior performance of our proposed method across Task I.

**Table 5:** Performance comparison of various studies of Task I.

Refs.	Physionet challenge ranking	Method	WAcc (train)	F-score (train)	WAcc (test)	F-score (test)
Reference ^26^	1	Mel-spectrogram and lightweight CNN	0.804	0.647	0.747	0.622
Reference ^27^	2	Parallel hidden semi-Markov models	0.817	0.645	0.758	0.625
Reference ^28^	3	Hierarchical multi-scale convolutional network	0.836	0.714	0.768	0.699
Reference ^44^	4	Hybrid Network	–	–	0.768	0.731
Reference ^45^	5	Multiple instance learning framework	0.828	0.568	0.734	0.544
This study	–	Deep features + RFE + RF	0.96	0.94	0.93	0.91

Furthermore, Table 6 contrasts our proposed method’s performance with other studies. This includes the top five ranked studies featured in the CinC/Physionet Challenge 2022 Task II. The results strongly indicate that the proposed method exhibits superior performance in terms of C_outcome_ compared to other methods. Notably, Reference^43^ demonstrated a higher AUC, utilizing both the Physionet 2016 and Physionet 2022 datasets. However, details about its performance in terms of the cost-based scoring metric (a crucial metric for Task II) and its performance in Task I were not reported. An interesting observation from these tables is that while the highest ranked teams in Task I, except reference ^27^, may not necessarily be among the top five teams in Task II. This underscores the efficacy and superiority of the proposed method in achieving exceptional performance across both tasks.

**Table 6:** Performance comparison of various studies of Task II.

Refs.	Physionet challenge ranking	Method	Dataset	C_outcome_ (train)	C_outcome_ (test)	AUC (test)
Reference ^27^	1	Parallel Hidden Semi-Markov Models	Physionet 2022	10,565	9257	0.693
Reference ^46^	2	Mel-spectrogram and CNN	Physionet 2022	10,458	9688	0.691
Reference ^47^	3	Handcrafted feature-based and deep learning Classifiers	Physionet 2022	6760	9420	0.663
Reference ^48^	4	Supervised contrastive network	Physionet 2022	7595	9479	0.614
Reference ^49^	5	Deep frequency-time domain features	Physionet 2022	8097	9493	0.66
Reference^43^	-	Bispectrum features- Vision Transformer	Physionet 2016 + Physionet 2022	-	-	0.98
This study	-	Deep features + RFE + SVM	Physionet 2022	4750	5290	0.78

## Discussion and conclusion

### Discussion

The results demonstrate that Stockwell effectively represents PCG signals by producing TFMs. This, in turn, enables Alexnet to extract meaningful features from TFMs for murmur identification. Notably, the robust performance of the Stockwell and Alexnet combination in representing PCG appears independent of the classifier algorithm. This is indicated in Table 1. Moreover, Tables 5 and 6 show that the proposed method outperformed other methods in both tasks of the 2022 Physionet challenge.

A noteworthy limitation of the present study is the constraint of analyzing 12.5-s segments of PCG signals, potentially restricting the scheme's performance, especially when PCG signals quality varies throughout the signal duration. To overcome this limitation, a potential enhancement involves integrating a signal quality assessment algorithm during preprocessing. This addition would facilitate the selection of high-quality 12.5-s segments for analysis. Alternatively, future studies could explore the utilization of various segments from each PCG record to ensure a more comprehensive analysis.

Additionally, despite the overall commendable performance of the proposed method in both tasks, it is essential to acknowledge that the accuracy of classifying normal from abnormal PCG signals (Task II) remains suboptimal and necessitates improvement (Table 4).

### Conclusion

This study introduced a novel approach that leverages the Stockwell transform and deep features extracted from PCG signals to significantly improve classification accuracy. The selection of the Stockwell transform was motivated by its superior time–frequency resolution compared to other methods, such as the wavelet transform, enabling a more detailed decomposition of PCG signal content. This study utilized all available records in the CinC/Physionet 2022 dataset for each patient. AlexNet's deep features provide a comprehensive description of each patient's heart condition. Given the abundance of deep features generated by AlexNet, RFE was employed to trim them down to 120 key features, which were then applied to the classifier. Impressively, the proposed method achieved a weighted accuracy of 93% for murmur detection (Task I) and a clinical outcome cost of 5290 for clinical outcome prediction (Task II). These results highlight the method's robust performance in both tasks when compared to high-ranking methods in the CinC/Physionet challenge 2022.

## Data Availability

The data that support the findings of this study was downloaded from https://physionet.org/content/circor-heart-sound/1.0.3/#files-panel . The dataset does not require specific permission for access and is publicly available for use.
